# Physiological demands and player characteristics in women’s futsal: a systematic review

**DOI:** 10.3389/fphys.2025.1642594

**Published:** 2025-08-01

**Authors:** Júlia Barreira, Karin Wunderlich, Ana Beatriz Chaves Vasconcelos Batista, João Eduardo Pereira da Silva Junior

**Affiliations:** Department of Sports Sciences, University of Campinas, Campinas, Brazil

**Keywords:** match demands, performance, game-analysis, physical capacities, neuromuscular profile

## Abstract

Women’s futsal is gaining increasing recognition in both the sporting world and academic research, driven by events such as the inaugural FIFA Women’s Futsal World Cup. This systematic review aimed to synthesize the current scientific literature on match demands and player characteristics in women’s futsal. Four key dimensions were analyzed: external and internal match loads (i), physiological, neuromuscular, and biochemical responses to competition (ii), and anthropometric (iii) and physical (iv) profiles of athletes across different competitive levels. The review followed PRISMA guidelines. Studies were included if they involved women practicing futsal at any competitive level, were peer-reviewed articles in English, Portuguese, or Spanish, and investigated variables related to match demands or player characteristics. Searches were conducted in SPORTDiscus, PubMed, Scopus, and Web of Science. The search returned 837 records; after removing duplicates and screening abstracts and full texts, 50 studies were included in the review. Methodological quality was assessed independently by two reviewers using a modified Downs and Black checklist. The findings indicate that women’s futsal imposes high physiological demands, especially in the first half of matches, with performance often declining in the second half. Elite players consistently show superior physical attributes—including higher VO_2_max, greater lean mass, and lower fat mass—compared to their lower-level counterparts. Evidence on neuromuscular characteristics suggests that agility and sprint capacity are particularly relevant to performance and responsive to training. Performance in the CMJ shows considerable variation among athletes of different competitive levels, highlighting the need for further research. Few studies have compared the performance of players in different positions, indicating a gap in the literature. Despite its growing popularity, the literature on women’s futsal remains limited and fragmented, with a predominance of cross-sectional designs, variability in measurement protocols, and a lack of data from official matches. To enhance training strategies and optimize athlete performance and health, future studies should adopt robust methodologies, include longitudinal designs, and explore the match demands and performance profiles of women’s players in real-world competitive contexts.

## 1 Introduction

Futsal has garnered increasing attention in the scientific literature in recent years (Moore et al., 2014; Spyrou et al., 2020). More specifically, women’s futsal has gained visibility and popularity, especially following the announcement of the inaugural FIFA Women’s Futsal World Cup, scheduled for 2025. This competition has drawn attention from both fans and professionals, as it requires high-level technical, tactical, and physical preparation. In response, many training programs have been developed to enhance athlete and team performance.

To better understand the demands of the game and the characteristics of the athletes, it is important to first consider the nature of futsal. Officially regulated by FIFA and commonly referred to as indoor soccer, futsal is played on a 40 × 20-m court in a five-a-side format, comprising four outfield players and a goalkeeper. It is a high-intensity intermittent sport that places substantial physical, technical, tactical, and psychological demands on players (Barbero-Alvarez et al., 2008). The game allows unlimited substitutions, enabling continuous player rotation to sustain intensity. Matches consist of two 20-min halves with a 10-min halftime break. However, since the clock is paused during stoppages (e.g., ball out-of-play, fouls, corners), total match duration may range from 70 to 90 min (Barbero-Alvarez et al., 2008; Doğramacı and Watsford, 2006). Teams are also allowed a 1-min time-out per half, which can be used strategically for tactical adjustments and recovery (Lovato and Barreira, 2025).

Review studies have made significant contributions to understanding the physical and physiological demands of futsal, as well as the most common tests used to assess attributes such as speed, agility, strength, and endurance. Naser et al. (2017), for instance, highlighted the current body of evidence on the physical, physiological, and technical-tactical demands of men’s futsal while identifying key gaps for future research. Spyrou et al. (2020) conducted a systematic review focusing on match demands and male athlete characteristics—including anthropometric, physiological, and neuromuscular aspects—across different competitive levels in futsal. Despite their valuable contributions, these reviews have primarily focused on the men’s game, with limited data available on women’s futsal. These gaps are especially critical when considering the biological, physiological, and cultural differences that may influence female athletes’ performance.

To date, only a few literature reviews have addressed women’s futsal specifically. These include studies focused on the sport within the Brazilian context (Barreira et al., 2018; Tamashiro and Galatti, 2018), as well as reviews investigating the epidemiology of injuries (Ruiz-Pérez et al., 2021) and the anthropometric profiles of players (Gómez-Campos et al., 2023). More recently, Barreira et al. (2024) conducted a comprehensive study offering an overview of the scientific output in this field. Their findings showed that most publications focus on strength training and physical conditioning. However, their review only briefly summarized the studies, reinforcing the need for more detailed and focused analyses to consolidate knowledge about physical preparation and other dimensions of women’s futsal.

Given this scenario, the present review aims to update and synthesize the current literature on match demands and the physical and neuromuscular characteristics of women’s futsal players across different competitive levels. This approach seeks to fill gaps in the literature and provide support for the development of more effective and context-specific training strategies for the sport.

## 2 Methods

### 2.1 Study design

The present study is a systematic review focused on the match demands and player characteristics in women’s futsal. The present review followed the guidelines established by the Preferred Reporting Items for Systematic Reviews and Meta-Analyses (PRISMA) (Moher et al., 2010). An initial search was carried out in databases including the Cochrane Database of Systematic Reviews, Scopus, SPORTDiscus, and Medline, and no existing or ongoing reviews addressing this topic were found. The review protocol was registered in the Open Science Framework, and ethical approval or informed consent was not required for this type of study.

### 2.2 Search strategy

This review was conducted using the following databases: SPORTDiscus, PubMed, Scopus, and Web of Science. The searches were carried out in March 2025, and records were identified through a combination of terms found in the title, abstract, and keywords within each database. The search strategy was structured using the terms “futsal,” “female,” and their synonyms. Truncations and Boolean operators were applied to optimize the search process. Specifically, the following terms were used: “futsal” AND “female” OR “woman” OR “women” OR “ladies” OR “lady”, following a procedure similar to that described by (Okholm et al., 2022).

### 2.3 Study selection

All retrieved records were exported to an online Excel spreadsheet to facilitate study selection. This spreadsheet was used to detect duplicates. Two reviewers (JB and JS) independently screened the titles and abstracts based on predefined inclusion and exclusion criteria. Disagreements regarding study inclusion were resolved through discussion in online meetings. In some cases, abstracts did not clearly indicate whether data for women’s futsal were presented separately from male or mixed teams, leading to uncertainty about inclusion. When this occurred, the studies were retained for full-text screening and re-evaluated thereafter. Selected articles were retrieved in full and stored in a shared folder to ensure that each was assessed in its entirety by at least two reviewers.

#### 2.3.1 Inclusion and exclusion criteria

Studies were included if they met the following criteria:

i) Involved women practicing futsal;
ii) Were published in English, Portuguese, or Spanish;
iii) Were peer-reviewed articles published in original scientific journals;
iv) Included participants at any skill level;
v) Investigated variables related to match demands or player characteristics.

The review included both cross-sectional and longitudinal studies. Year of publication was not used as a selection criterion.

Studies were excluded if:

i) The gender of participants was not identified;
ii) They focused on mixed-gender futsal teams;
iii) Results were combined with those of male athletes;
iv) Results were combined with data from other sports;
v) The methodological quality assessment score was ≤8.

For studies investigating anthropometric characteristics, only those that included at least two anthropometric measures were included. This was necessary because some studies reported only the body mass index (BMI).

### 2.4 Data extraction

The software PlotDigitizer was used to extract data presented in figures when necessary. Variables collected included: authorship, year of publication, country/location of the study, participant population, sample size, and competitive level of the athletes. Competitive level classification was defined as follows (Okholm et al., 2022):

• Recreational–Competitive play at any amateur level;
• College–Players competing at the college or university level;
• High level–Players competing in national (second or third division), highly trained, high-performance, or semi-professional teams;
• Elite–Players competing in the top national league, international competitions, or national teams.

External load measures were determined after full-text analysis, based on the variables reported in each study. Key parameters included: total distance covered, relative speed, distance covered at high speed, high-intensity metabolic distance, maximum sprint speed, and the number of accelerations and decelerations performed during matches. Similarly, internal load was defined post hoc, based on the data available in the studies. Notably, all studies used heart rate as the sole indicator of internal load, reporting both absolute and relative values throughout match play.

Regarding player characteristics, the data collection began with a comprehensive inventory of all physical tests used across studies. From this analysis, we summarized in a table the physical performance tests most commonly reported in systematic reviews on football and futsal (Datson et al., 2014; Spyrou et al., 2020; Hall et al., 2024), and which were also widely used in women’s futsal research. Nevertheless, other physical tests are also discussed throughout the text, even if not summarized in the main table.

For anthropometric data, we prioritized information on body mass, lean mass, and fat mass. When studies presented comparisons by playing position, data were organized accordingly to allow for a more segmented analysis. This approach enabled more precise interpretations of match demands and player characteristics according to positional roles in women’s futsal.

In studies that reported multiple groups or data collected at different time points, we prioritized data from the control group or pre-intervention phase. When uncertainty arose regarding which data to extract, we opted to include all relevant available information.

### 2.5 Methodological quality assessment

Using the modified Downs and Black scale (Downs and Black, 1998), two researchers (AB and KC) independently evaluated the methodological quality of the studies and any disputes were resolved by a third reviewer (JB). Of the 27 criteria of the original scale, 12 were applied in this study, as observed in previously published research with similar designs (Spyrou et al., 2020; Whitehead et al., 2018).

## 3 Results and discussion

**FIGURE 1:**
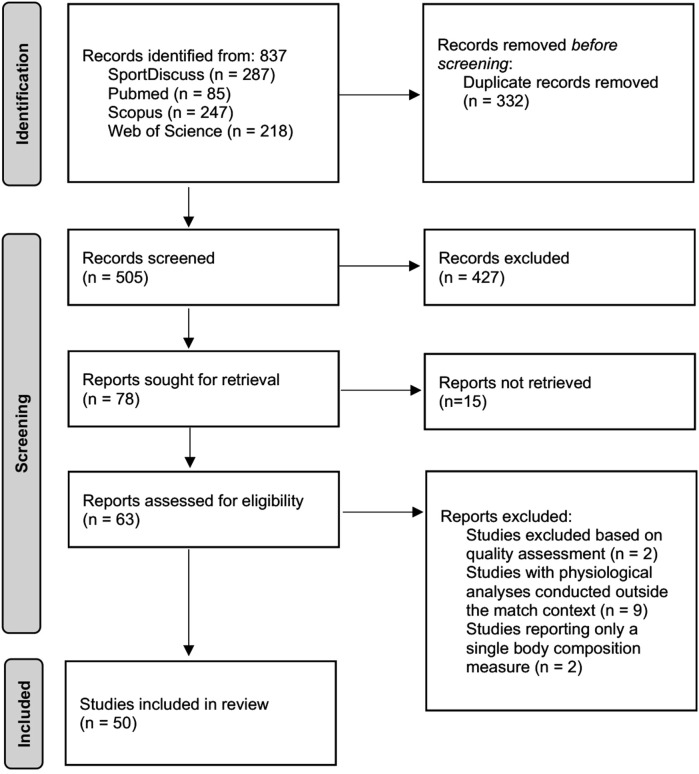
Flow chart of the study inclusion process.

Figure 1 presents the PRISMA flow diagram of the search and study selection process. The database search initially returned 837 citations, from which 332 duplicate records were removed. The remaining 505 articles were screened based on their titles and abstracts according to the predefined inclusion and exclusion criteria. Of these, 78 met the criteria for full-text review. Following the full-text analysis, a total of 50 articles were included in the final review.

### 3.1 External and internal match load

Understanding both external and internal load during matches is essential to comprehend the physiological and biomechanical demands placed on athletes, informing more effective training interventions (Carminati et al., 2015). One of our main findings is the limited number of studies addressing the specific demands of women’s futsal matches. Only three studies were found that examined external load during matches (Beato et al., 2017; Vieira et al., 2021; Oliva-Lozano et al., 2023).

Beato et al. (2017) examined external load in friendly matches played on synthetic turf (38 × 18 m) by second-division Italian players. The study reported statistically significant reductions from the first to the second half in total distance covered (1,424 ± 114 m vs. 1,313 ± 113 m, p < 0.05), relative velocity (70 ± 6 m/min vs. 64 ± 6 m/min, p < 0.05), high-speed running distance (28 ± 16 m vs. 22 ± 19 m, p < 0.05), and high metabolic distance (80 ± 29 m vs. 69 ± 28 m, p < 0.05). Similarly, Vieira et al. (2021) investigated first-division Brazilian players and found significant reductions in total distance covered (1,659 ± 100 m vs. 1,547 ± 102 m, p < 0.01) and sprint speed (31.2 ± 5.4 km/h vs. 26.3 ± 6.4 km/h, p < 0.01) from the first to the second half. Most time was spent at moderate intensities (6.1–12 km/h). Oliva-Lozano et al. (2023) focused on official matches in Spain’s first division and observed a decline in high-intensity running distance from the first to the second half (14.7 ± 4.7 m/min vs. 13.1 ± 4.7 m/min, p < 0.01), with no significant change in the number of accelerations and decelerations.

Together, these studies indicate that women’s futsal players exhibit a decline in physical performance during the second half of matches. Comparable patterns have been observed in men’s futsal, where time spent walking or standing increases in the second half, accompanied by reductions in medium-to-high velocity running and sprint distances, although total sprint counts remain relatively unchanged (Bueno et al., 2014; Milioni et al., 2016). Reductions in total distance, high-intensity running, and sprint speed may reflect energy depletion and fatigue accumulation over the course of a game.

Notably, current studies in women’s futsal have not reported the total distance covered across entire matches, presenting data only by half, and no comparisons have been made across competitive levels (e.g., professional vs. semi-professional). The predominance of cross-sectional studies and research conducted in simulated or friendly match settings represents also a significant limitation. In championship matches, factors such as performance pressure, more complex tactical strategies, officiating decisions, and the opponent’s competitive level can substantially influence players’ responses in terms of both internal load and performance. The absence of these elements in simulated settings compromises the ecological validity of the findings and limits their generalizability to high-performance environments. Therefore, future research should prioritize longitudinal designs and data collection during official matches, incorporating tools such as players tracking, accelerometry, and contextual analysis (e.g., scoreline, match time, opponent level) to more accurately reflect the complexity of the game and its impact on female futsal players.

Regarding the internal load, heart rate (HR) monitoring was the most commonly used method to assess internal load during futsal matches. This review identified four studies that analyzed the HR responses of women’s futsal players in both official and simulated games (Oliva-Lozano et al., 2023; Carminatti et al., 2015; Kassiano et al., 2019; Apriantono et al., 2021).

Oliva-Lozano et al. (2023) examined first-division Spanish players and reported significant differences in the distribution of time spent across various HR zones between halves. Specifically, players spent a higher percentage of time in the HR60%–70% (2.8% ± 4.3% vs. 4.7% ± 8.5%, p < 0.01), HR70%–80% (10.2% ± 9.7% vs. 13.6% ± 13.1%, p < 0.01), and HR80%–90% (31.3% ± 19.6% vs. 35.6% ± 17.1%, p < 0.01) ranges during the second half, while the time spent in the HR90%–95% (43.6% ± 22.6% vs. 36.2% ± 21.1%, p < 0.01) and HR>95% (10.6% ± 14.3% vs. 7.2% ± 10.4%, p < 0.01) zones was significantly greater during the first half.

Carminatti et al. (2015) assessed Brazilian professional players over five matches—three friendly and two officials—and found a slight reduction in average HR from the first (91.6% ± 3.8% HRmax) to the second half (90.5% ± 3.5% HRmax, p = 0.034). Mean HR during official games was 91.1% ± 2.4% HRmax, compared to 90.0% ± 4.1% in simulated matches, although no statistical comparison was conducted between match types. Similarly, Kassiano et al. (2019) analyzed semi-professional university players and observed a decrease in mean HR from the first (173.8 ± 9.7 bpm) to the second half (169.0 ± 9.3 bpm), though no statistical analysis was reported. Apriantono et al. (2021) provided average HR values from official matches involving three different teams but did not compare between halves. Reported mean HRs were: Team A (168.1 ± 1.7 bpm vs. 171.4 ± 2.0 bpm), Team B (166.5 ± 2.9 bpm vs. 168.7 ± 3.0 bpm), and Team C (166.9 ± 2.4 bpm vs. 169.3 ± 1.3 bpm), respectively for the first and second halves.

The findings suggest a trend of reduced physiological intensity in the second half of futsal matches, as evidenced by the shift in HR distribution toward lower zones. Players spent less time in the highest intensity zones (HR > 90%) and more time in moderate zones (60%–90%) during the second half. This indicates a progressive decline in internal load, likely due to fatigue accumulation and reduced ability to sustain high-intensity efforts. From a training perspective, these results highlight the need to improve players’ ability to maintain high physiological intensity throughout the entire match. Coaches should prioritize conditioning strategies that enhance high-intensity aerobic and anaerobic capacity.

Despite these insights, the current body of literature is limited by the predominance of simulated or friendly matches and by the narrow focus on HR alone as an internal load marker. This limits the generalizability of findings to elite competition settings. Only three studies have explored additional physiological and biochemical markers. Santos et al. (2018) investigated salivary cortisol and creatine kinase (CK) levels in 14 players over four matches, finding no statistically significant changes from baseline to post-match. Conversely, Kassiano et al. (2019) reported substantial increases in blood lactate concentrations, rising from 2.1 ± 0.3 to 6.3 ± 2.3 mmol/L by the end of the first half, and remaining elevated after the second half (5.4 ± 1.3 mmol/L). More recently, Souglis et al. (2023) analyzed the time-course effects of a futsal match on performance (CMJ and sprint), oxidative stress (PC and TBARS), and muscle damage markers (LDH and CK), as well as inflammatory (IL-6, TNF-α, WBC, CRP, FIB, HCY) and antioxidant responses of women’s futsal players. The study showed that the match induced short/mid-term changes in performance, inflammation, oxidative stress, and muscle damage markers for about 72 h–96 h post-match.

Although few studies have investigated physiological and biochemical markers in women’s futsal, the available evidence offers valuable insights for training and recovery management. Elevated levels of lactate and CK following match play indicate significant metabolic and muscular stress, which may persist for 48–96 h, as reported in recent research (e.g., Souglis et al., 2023). These findings suggest that players may require extended recovery periods after high-intensity matches. From a practical standpoint, post-match monitoring of biochemical markers can guide individualized recovery strategies, helping to prevent overtraining and reduce the risk of injury—particularly during congested competitive schedules. High-load training sessions, especially those involving substantial neuromuscular or metabolic demands, should be strategically planned, allowing for at least 48–72 h of recovery when elevated CK or inflammatory markers are observed. Strength and conditioning professionals are encouraged to implement routine screening protocols to monitor these indicators and adjust training loads accordingly. Incorporating such data into periodization plans may improve athlete readiness, enhance performance, and support long-term health.

### 3.2 Anthropometrics

Anthropometric characteristics (i.e., height, body mass, and body composition) are important components for futsal players, as they are highly correlated with the physical capacities required for match performance (Lago-Fuentes et al., 2020; Hoffmann et al., 2021; Pinho et al., 2022; Castillo et al., 2022a). Several studies have investigated the anthropometric profiles of women’s futsal players, with the database search including 15 articles (Queiroga et al., 2005; Queiroga et al., 2018; Queiroga et al., 2019; Rocha et al., 2013; Lago-Fuentes et al., 2020; Ferreira et al., 2020; Castillo et al., 2022a; Castillo et al., 2022b; Pinho et al., 2022; Hoffmann et al., 2021; Ari, 2020; Campos et al., 2016; Weber et al., 2024). Overall, futsal players tend to have an average body mass ranging from 55 to 65 kg and body fat percentages between 11% and 26%, depending on the competitive level and assessment method employed.

Studies comparing players by playing positions have revealed significant differences in anthropometric characteristics (Queiroga et al., 2005; Rocha et al., 2013; Castillo et al., 2022a). Queiroga et al. (2005) reported higher body mass, BMI, and body fat percentage among goalkeepers (63.9 ± 8.3 kg; 23.5 ± 2.3 kg/m^2^; 26.7% ± 4.8%, respectively) compared to outfield players. Similar results were observed by Rocha et al. (2013), who found greater body mass in goalkeepers (65.9 ± 3.43 kg) than in wingers (55.12 ± 5.47 kg), although no statistically significant differences in body fat percentage were reported between positions. In Castillo et al. (2022a), the pivot and goalkeeper positions had the highest body mass (65.43 ± 6.51 kg and 64.31 ± 7.47 kg, respectively), with significant differences compared to other positions such as forwards (58.63 ± 7.69 kg), winger-pivots (60.36 ± 3.55 kg), and wingers (55.65 ± 4.77 kg). However, body fat percentage did not significantly differ across positions.

Some studies investigated whether variables such as starter status, birth period, and generational cohort influence players’ anthropometric and body composition profiles. Regarding starter status, Queiroga et al. (2018) evaluated 115 athletes from 10 professional Brazilian teams and found a small but significant difference in body fat percentage between starters (21.3% ± 5.1%) and non-starters (23.2% ± 5.2%), with no differences in body mass (57.8 ± 9.1 kg) or lean mass (45.4 ± 4.5 kg). Ferreira et al. (2020), using the same sample, grouped players by birth quartiles and found no significant differences in body mass (58.6 ± 7.6 kg), lean mass (45.4 ± 4.5 kg), or fat mass (22.1% ± 5.2%) across groups, suggesting that birth period had no substantial impact on the body composition of these athletes. Additionally, Queiroga et al. (2019) conducted a longitudinal analysis comparing two generations of elite-level athletes: one group of 112 players evaluated in 2001 and another with 115 players evaluated in 2011. The authors found no significant differences in body mass (58.5 ± 7.3 kg), lean mass (45.0 ± 4.3 kg), or body fat percentage (22.4% ± 5.1%) between the two cohorts, indicating relative stability in these anthropometric variables over a decade. However, the same research group recently reported a significant difference in the body fat percentage of athletes assessed in the years 2001 and 2011 compared to 2021, with a marked reduction to 19.0% ± 5.1%, highlighting the potential impact of training and dietary changes driven by the sport’s development (Weber et al., 2024).

Although the literature has explored the body composition of players across different competitive levels, no studies have directly compared their characteristics. In the university context, Pinho et al. (2022) evaluated 19 regional-level players, using skinfold measurements to estimate body composition, reporting mean body mass of 63.3 ± 12.06 kg, body fat percentage of 26.3% ± 3.7%, and lean mass of 46.1 ± 4.9 kg. Similarly, Hoffmann et al. (2021) analyzed 19 university players using skinfolds and reported a mean body mass of 61.9 ± 11.2 kg and body fat percentage of 25.2% ± 4.9%, representing the highest fat percentages among all studies included in this review.

In contrast, elite players typically exhibit lower body fat percentages. In a study by Castillo et al. (2022b), 12 players from Spain’s First Division were assessed using skinfolds, yielding a mean body mass of 59.79 ± 6.36 kg, body fat percentage of 21.03% ± 3.05%, and muscle mass of 22.95 ± 5.80 kg. Lago-Fuentes et al. (2020) evaluated 14 players from the Spanish Second Division (mean age = 21.6 ± 3.6 years), also using skinfolds, and reported a body fat percentage of 20.12% ± 3.46%, muscle mass of 44.89% ± 3.31%, and bone mass component of 14.1% ± 0.85%. Slightly lower fat mass values were reported in a study on players from the Brazilian national team (Rocha et al., 2013), with skinfold-derived body fat percentages of 12.10% ± 1.65% for pivots and 16.48% ± 1.51% for goalkeepers. These findings suggest that while elite players tend to present lower fat percentages and higher lean mass, these values may vary substantially depending on the evaluation method, sample characteristics, and competitive level. Therefore, more standardized studies are needed to enable consistent comparisons across competitive contexts.

Additionally, methodological aspects must be considered, as they significantly affect body composition outcomes. Castillo et al. (2022a) demonstrated considerable variation in body fat estimates when different equations were applied to the same sample: Rocha (16.22% ± 1.42%), Withers (11.93% ± 4.41%), Faulkner (13.81% ± 2.43%), and Carter (11.56% ± 12.44%). These discrepancies underscore the need for studies that systematically compare predictive equations to identify those offering the highest accuracy and applicability for women’s futsal players. It is also worth noting that most studies employed skinfold measurements to assess body composition. Only Campos et al. (2016) used dual-energy X-ray absorptiometry (DXA) in a sample of recreational-level players, reporting 16.8 ± 3.8 kg of fat mass and 38.9 ± 3.4 kg of fat-free mass. Given the scarcity of studies using DXA, we emphasize the need for further research employing this method to explore the anthropometric profiles of athletes across different competitive levels.

### 3.3 Physiological characteristics

The aerobic energy system plays a fundamental role during futsal matches, as it has been shown to contribute to recovery after high-intensity efforts and to differentiate athletes according to their competitive level (Alvarez et al., 2009). During futsal matches, players cover approximately 3 km, performing repeated sprints, accelerations, and decelerations, often with short recovery intervals (Beato et al., 2017; Vieira et al., 2021). This match demands reinforces the importance of a well-developed aerobic system.

Upon reviewing the literature, nine studies (Pacheco et al., 2009; Karahan, 2012; Barbero-Alvarez et al., 2015; Hoffmann et al., 2021; Nabo et al., 2021; Costa et al., 2021; Colantonio et al., 2020; Pinho et al., 2022; Vieira et al., 2021; Rocha et al., 2013) analyzed the maximal oxygen consumption (VO_2_max) in women’s futsal players. VO_2_max values vary according to competitive level, season period, and testing protocol. For instance, studies using progressive treadmill protocols reported values ranging from 36.9 to 52.2 mL kg^-1^·min^-1^ in Venezuelan national team athletes (Barbero-Alvarez et al., 2015), 34–59 mL kg^-1^·min^-1^ in Brazilian university players (Hoffmann et al., 2021), and 48.9 ± 4.1 mL kg^-1^·min^-1^ in elite players from the Brazilian first division (Vieira et al., 2021). Although few investigations directly compare athletes of different competitive levels, data suggest that players competing in national teams or high-performance leagues tend to present higher or upper-range VO_2_max values (44–57 mL kg^-1^·min^-1^), compared to university or regional-level athletes, who generally exhibit averages between 39 and 44 mL kg^-1^·min^-1^. These findings indicate a positive influence of competitive level on aerobic capacity development.

Additionally, variations in VO_2_max values were observed across the season or following specific training interventions. For example, significant increases were noted from the preparatory to the competitive period (43.8–47.6 mL kg^-1^·min^-1^) (Pacheco et al., 2009), as well as after interval or continuous training protocols (39.5–43.6 mL kg^-1^·min^-1^ and 40.4 ± 5.2 to 42.0 ± 9.2 mL kg^-1^·min^-1^) (Karahan, 2012; Colantonio et al., 2020). Physiological factors, such as the menstrual cycle, also appear to influence this variable. Nabo et al. (2021) identified significant differences across menstrual cycle phases, with higher values in the luteal phase (41.2 mL kg^-1^·min^-1^) compared to the follicular phase (39.0 mL kg^-1^·min^-1^). These findings underscore the importance of considering both competitive context and timing of assessment when interpreting VO_2_max results in women’s futsal players.

Field tests have proven to be valuable tools for assessing aerobic endurance in futsal players (Parnow et al., 2024). The scientific literature reports various protocols to measure this capacity in futsal players, including the Yo-Yo Intermittent Recovery Test (levels 1 and 2), Shuttle Run Test, Futsal Intermittent Endurance Test (FIET), Running-based Anaerobic Sprint Test (RAST), the 30–15 Intermittent Fitness Test, and the Carminatti test (Spyrou et al., 2020). Among these protocols, the Yo-Yo Intermittent Recovery Test Level 1 (Yo-Yo IR1) stands out as the most widely adopted in women’s futsal players (Baskaya et al., 2018; Beato et al., 2017; Cuaspa Burgos and Sanchez, 2022; Castillo et al., 2022a; Clemente et al., 2018; Costa et al., 2021; Gomes et al., 2021; Pinto et al., 2021; Pinho et al., 2022; Santos et al., 2020) — as summarized in Table 1.

**TABLE 1:** Summary of studies that used the Yo-Yo Intermittent Recovery Test Level 1 (Yo-Yo IR1) to assess female futsal players.

Study	Participants (n)	Level	Yoyo level 1 (m)
Baskaya et al. (2018)	30	College	924.6 ± 263.6
Beato et al. (2017)	16	High Level	920 ± 164
Cuaspa Burgos and Sanchez, 2022	19	High Level	757.9 ± 249.9
Castillo et al. (2022b)	12	Elite	1,120 ± 336.4
Clemente et al. (2018)	10	High Level	1,271.8 ± 231.1
Costa et al. (2021)	13	Recreational	341.5 ± 84.2
Gomes et al. (2021)	13	College	302.2 ± 100.2
Pinto et al. (2021)	16	High level	712 ± 247.5
Pinho et al. (2022)	19	High Level	400 ± 106.9
Santos et al. (2020)	13	Not informed	350.8 ± 88.2

Data are presented as mean ± standard deviation.

The analysis of studies reveals high variability in distances covered, highlighting differences primarily related to competitive level. Recreational or university-level players generally show lower performance, with distances ranging from 300 to 400 m in the Yo-Yo IR1 (Costa et al., 2021; Gomes et al., 2021; Santos et al., 2020). The latter, although not specifying the participants’ competitive level, reported a mean distance of 350.8 ± 88.2 m—a value consistent with lower-level samples. High-level players, on the other hand, often exceed 800 m (Baskaya et al., 2018; Beato et al., 2017; Castillo et al., 2022a), sometimes reaching values close to 1,270 m (Clemente et al., 2018). In one study, even university athletes reached ∼924 m, suggesting that competitive level does not always outweigh factors such as training history or the testing protocol used (Baskaya et al., 2018).

The available evidence highlights the relevance of aerobic capacity for women’s futsal players, particularly due to the sport’s intermittent and high-intensity nature. Despite methodological variations across studies—including differences in testing protocols, competitive levels, and training periods—VO_2_max values tend to be higher in elite or national-level athletes, suggesting a positive association between aerobic fitness and performance level. The frequent use of field tests, especially the Yo-Yo Intermittent Recovery Test Level 1, reinforces their practical utility for monitoring aerobic endurance in sport-specific contexts. However, the observed variability in test results also indicates that factors such as training history, testing conditions, and physiological states (e.g., menstrual cycle) should be carefully considered when interpreting outcomes. Taken together, these findings support the inclusion of targeted aerobic training and regular monitoring in the preparation of women’s futsal athletes to enhance performance and optimize recovery.

### 3.4 Neuromuscular characteristics

Physical tests play a key role in monitoring, evaluating, and prescribing training for athletes in this sport. These procedures allow strength and conditioning professionals to assess athletes’ progress throughout the season, make necessary adjustments to training programs, and guide more effective performance interventions (Turner et al., 2011). Table 2 shows a summary of studies that used physical tests to analyze the neuromuscular characteristics of women’s futsal players.

**TABLE 2:** Summary of neuromuscular characteristics of women’s futsal players.

Study	Participants (n)	Level	Flexibility	Agility			Sprinting			Jump (cm)
			Sit and reach (cm)	Illinois agility test (s)	Shutle run (s)	T-test (s)	10 m (s)	20 m (s)	30 m (s)	CMJ (cm)
Alvares et al. (2017)	17	High Level	30.4 + 7.7	NR	10.6 ± 0.4	NR	NR	NR	NR	NR
Ari. (2020)	17	College	NR	NR	NR	NR	1.94 ± 0.15	NR	5.14 ± 0.27	34.75 ± 4.51
Baskaya et al. (2018)	30	College	24.70 ± 7.40	17.23 ± 0.82	NR	NR	1.62 ± 0.081	NR	4,31 ± 0.358	23.22 ± 2.20
Beato et al. (2017)	16	High Level	NR	NR	NR	NR	NR	NR	NR	NR
Cuaspa Burgos and Sanchez. (2022)	19	High Level	NR	NR	NR	NR	NR	NR	NR	NR
Castillo et al. (2022b)	12	Elite	NR	NR	NR	11.01 ± 0.31	NR	NR	NR	31.47 ± 2.82
Ceylan et al. (2014)	10	College	29.63 ± 10.06	NR	NR	NR	NR	NR	6.25 ± 0.55	NR
Clemente et al. (2018)	10	High Level	NR	NR	NR	NR	NR	NR	NR	NR
Costa et al. (2021)	13	Recreational	NR	NR	NR	NR	NR	NR	NR	NR
David et al. (2022)	14	High Level	NR	NR	NR	12.08 ± 0.49	NR	4.00 ± 0.17	NR	NR
Doewes et al. (2022)	20	Not informed	NR	19.90 ± 0.71	NR	NR	NR	3.76 ± 0.21	5.63 ± 0.17	NR
Fiorante and Pellegrinotti. (2018)	13	Recreational	25.71 ± 5.15	NR	NR	NR	NR	4.86 ± 0.45	NR	NR
Gomes et al. (2021)	13	College	NR	NR	NR	NR	NR	NR	NR	28.2 ± 2.3
kassiano et al. (2019)	6	College	NR	NR	NR	NR	NR	NR	NR	41.9 ± 2.9
Lago-Fuentes et al. (202)	14	Elite	NR	NR	NR	NR	CTS: 1.98 ± 0.05 CTU: 1.94 ± 0.08	NR	NR	CTS: 25.24 ± 3.53 CTU: 26.78 ± 2.77
Moreira et al. (2013)	45	High level	NR	NR	Sub 15:12.4 ± 0.6 Sub 17:12.1 ± 0.5 Sub 19:12.0 ± 0.6 Adult:12.1 ± 0.8	NR	NR	NR	NR	NR
Patti et al. (2022)	15	Elite	NR	NR	NR	11.59 ± 1.34	NR	NR	NR	NR
Pinto et al. (2021)	16	High level	NR	NR	NR	NR	NR	NR	NR	NR
Pinho et al. (2022)	19	High Level	NR	NR	NR	NR	NR	NR	NR	NR
Ramos-Campo et al. (2016)	27	Elite (n = 14) High level (n = 13)	NR	NR	NR	NR	NR	NR	Elite 4.9 ± 0.2 High level 5.0 ± 0.2	Elite: 26.7 ± 0.3 High level: 24.3 ± 0.3
Rocha et al. (2015)	12	Elite	36.8 ± 5.7	NR	9.9 ± 0.3	NR	NR	NR	NR	NR
Ruiz-Pérez et al. (2023)	22	Elite	NR	16.97 ± 0.60	NR	NR	1.99 ± 0.14	NR	NR	28.0 ± 3.0
Santos et al. (2020)	13	Not informed	NR	19.6 ± 0.7	NR	NR	NR	NR	NR	18.5 ± 1.7
Silva et al. (2017)	10	College	24.9 ± 3,7	NR	NR	NR	NR	NR	NR	NR
Ünveren. (2015)	35	College	NR	1.99 ± 0.55	NR	NR	1.60 ± 0.11	2.98 ± 0.20	4.16 ± 0.39	NR
Yanez et al. (2023)	14	College	NR	NR	NR	NR	NR	NR	NR	29.02 ± 5.25
Lucas et al. (2024)	25	College	NR	NR	NR	NR	NR	3.28 ± 0.27	NR	25.69 ± 4.58

Legend: NR: not reported; CTS: group that performed core strengthening exercises on a stable surface; CTU: group that performed the same exercises on an unstable surface; values are expressed as mean ± standard deviation (mean ± SD).

### 3.5 Jumping ability

In women’s futsal, various studies have highlighted the importance of muscular power for both physical and technical performance during matches, given the high frequency of intense actions such as sprints, changes of direction, and jumps (Caetano et al., 2015; Ribeiro et al., 2016). For this reason, the countermovement jump (CMJ) has been widely adopted as a neuromuscular performance indicator. Studies have examined athletes across different competitive levels, training contexts, and physical conditions to describe and better understand vertical jump profiles in women’s futsal (Gorostiaga et al., 2005; Ramos-Campo et al., 2016; Castillo et al., 2022a; Gomes et al., 2021). Specifically, ten studies (Ari, 2020; Baskaya et al., 2018; Castillo et al., 2022a; Gomes et al., 2021; Lago-Fuentes et al., 2020; Ramos-Campo et al., 2016; Ruiz-Pérez et al., 2023; Santos et al., 2020; Yanez et al., 2023; Lucas et al., 2024).

The data from these studies indicate that the average CMJ height among women’s futsal players ranges from approximately 18.5 cm–31.4 cm, depending on competitive level and assessment methods (Santos et al., 2020; Castillo et al., 2022a; Ramos-Campo et al., 2016). For instance, Ari (2020) reported a mean jump height of 34.75 ± 4.51 cm in university players, while Baskaya et al. (2018) and Lucas et al. (2024) found an average of 23.22 ± 2.20 cm and 25.69 ± 4.58 cm, respectively, among athletes at the same level. These variations may be attributed to differences in physical conditioning, testing protocols, or measurement devices. For high-performance players, Ramos-Campo et al. (2016) reported no statistically significant differences in CMJ performance between elite and sub-elite players (26.7 ± 0.3 cm vs. 24.3 ± 0.3 cm), suggesting that this measure alone may not be sufficient to distinguish between these levels within the same sport.

Regarding associations with other physical capacities, studies by Ari (2020) and Gomes et al. (2021) found moderate positive correlations between CMJ performance and variables such as speed, agility, and overall quality of life. Ari (2020), for example, observed that players with higher jump heights also achieved better times in 30 m sprint and agility tests, reinforcing the role of lower-limb power in overall performance.

Jump tests have also been employed to monitor neuromuscular fatigue in various settings. Santos et al. (2020) and Yanez et al. (2023) found that the CMJ is sensitive to neuromuscular fatigue or accumulated effort following matches or simulated protocols. These findings support the use of the CMJ not only as a power assessment tool but also as a physical monitoring strategy throughout the season, training sessions, and matches.

Other studies have also examined differences across various jump height assessment protocols (Lago-Fuentes et al., 2020; Castillo et al., 2022b; Ramos-Campo et al., 2016; Ruiz-Pérez et al., 2023). In the study by Castillo et al. (2022b), three different jump tests were used: the Squat Jump (SJ), the Countermovement Jump (CMJ), and the Abalakov Jump (ABK). The mean results were: SJ = 28.95 ± 2.57 cm; CMJ = 31.47 ± 2.82 cm; and ABK = 33.60 ± 3.75 cm. Interestingly, only the ABK showed a moderate correlation with player height (r ≈ 0.60), suggesting that the CMJ, by restricting arm use, may serve as a more accurate indicator of lower-limb power without interference from morphological traits such as stature. Ramos-Campo et al. (2016) reported significantly lower CMJ and values for elite players and sub-elite (CMJ: 26.7 ± 0.3 vs. 24.3 ± 0.3 cm; SJ: 26.1 ± 0.4 vs. 24.2 ± 0.3 cm). They noted that the timing of the season (early pre-season) and similar training routines across groups may have limited performance differences. Similarly, Lago-Fuentes et al. (2020) found low CMJ values (∼25–26 cm) when evaluating core training on unstable surfaces, with slight post-training improvements that were not statistically significant.

In general, vertical jump is recognized as a relevant skill in futsal. Despite variations in jump protocols—such as arm use, equipment type, and number of attempts—the CMJ stands out as a reliable and practical indicator of lower-body power. Although players from different competitive levels have been studied, no research to date has compared these levels within a single study, nor have differences across playing positions been explored. Therefore, future studies could aim to address these gaps in the literature.

### 3.6 Sprinting ability

Speed is closely related to the ability to perform rapid movements and respond efficiently to game demands, such as offensive and defensive transitions (Doewes et al., 2022; Ramos-Campo et al., 2016). In futsal, it is expressed through quick reaction times and effective change of direction (Doewes et al., 2022). To assess and train speed in futsal, standardized sprint tests over short distances—typically between 5 and 30 m—are commonly used and several studies have assessed sprint performance over these distances using different protocols and samples (Ari, 2020; Baskaya et al., 2018; Ceylan et al., 2014; David et al., 2022; Doewes et al., 2022; Fiorante and Pellegrinotti, 2018; Lago-Fuentes et al., 2020; Ramos-Campo et al., 2016; Ruiz-Pérez et al., 2023; Ünveren, 2015; Lucas et al., 2024).

Most studies have investigated college-level players, reporting 30 m sprint times ranging from 4.3 to 6.2 s (Baskaya et al., 2018; Ceylan et al., 2014). Only one study compared players from different competitive levels, showing no significant differences in 30 m sprint performance between elite and sub-elite futsal players (elite: 4.9 ± 0.2 s; sub-elite: 5.0 ± 0.2 s) (Ramos-Campo et al., 2016), suggesting that shorter distances may be more sensitive for detecting speed-related performance differences in competitive settings. Therefore, future studies should explore differences in sprint performance across competitive levels and tactical roles.

Regarding training-induced adaptations, David et al. (2022) and Lago-Fuentes et al. (2020) demonstrated that well-structured interventions—such as training macrocycles or core strengthening programs—can lead to significant improvements in sprint performance. For example, David et al. reported a reduction in 20 m sprint time from 4.00 ± 0.17 s (pre-season) to 3.74 ± 0.21 s (post-season). In Lago-Fuentes et al. (2020), 6 weeks of core training resulted in up to a 5% improvement in 10 m sprint performance.

The studies included in this review also indicated that futsal players tend to outperform football players in sprint tests. For instance, Baskaya et al. (2018) found that futsal players recorded significantly faster sprint times over 10 m (1.62 ± 0.08 s) and 30 m (4.31 ± 0.35 s) compared to football players. Similarly, Ünveren (2015) reported superior sprint performance in futsal players over 10 m (1.60 ± 0.11 s), 20 m (2.98 ± 0.20 s), and 30 m (4.16 ± 0.39 s), with football players showing slower times across all distances. According to Naser et al. (2017), futsal matches tend to be more intense than football matches, partly due to the allowance of unlimited substitutions. This feature enables players to sustain high-intensity efforts for longer periods throughout the game. As a result, futsal is considered a predominantly anaerobic sport, characterized by repeated sprints and a greater proportion of high-intensity actions compared to football and other intermittent sports.

Overall, the reviewed literature highlights the importance of sprint performance as a key physical attribute in futsal, particularly over short distances. Futsal players not only outperform football players in various sprint tests but also show responsiveness to specific training interventions aimed at improving speed. While current evidence suggests that sprint capacity may be similar between elite and sub-elite players over longer distances, future research should further explore sprint performance across different competitive levels and playing positions, as well as investigate which distances are most sensitive to performance differences. Understanding these nuances may support more targeted training strategies in women’s futsal.

### 3.7 Agility

Agility is defined as the ability to perform rapid body movements involving changes in velocity or direction in response to a stimulus (Benvenuti et al., 2010). In women’s futsal, this physical quality is particularly critical due to the smaller court dimensions and the game’s high intensity. Players are constantly accelerating, decelerating, and changing direction in tight spaces under defensive pressure, requiring high levels of agility and quick decision-making (Doewes et al., 2022; Ramos-Campo et al., 2016).

Various agility tests have been employed in the literature, including both standardized and futsal-specific protocols. Among the most widely used change of direction (COD) tests is the Illinois Agility Test and the T-Test. Additionally, short-distance shuttle run protocols such as the 4 × 10 m test are often employed. This review included results from these tests, as the most reported in the selected studies.

In the Illinois Agility Test, a longer course with multiple directional changes, typical times for university and regional-level players ranged from ∼17 to 20 s. In the 4 × 10 m shuttle run, characterized by shorter sprints and rapid directional changes, performance varied by age group and competitive level. For instance, Moreira et al. (2013) reported times of 12.4 s (U-15), 12.1 s (U-17), 12.0 s (U-19), and 12.0 s (adults). Elite players, however, demonstrated superior performance, with Rocha et al. (2015) reporting times close to 9 s.

Similarly, results from the T-Test revealed performance differences based on competitive level. Elite players evaluated by Castillo et al. (2022b) and Patti et al. (2022) recorded average times of ∼11 s. In contrast, players from the Campeonato Gaúcho de Futsal Feminino (David et al., 2022), a Brazilian regional championship, showed a higher average of ∼12 s. These findings suggest that the T-Test is sensitive to performance differences in women’s futsal, reflecting athletes’ ability to execute quick, multidirectional movements. The superior results of elite players may be attributed to higher training volume and quality, better neuromuscular condition and more efficient movement patterns.

When choosing assessment protocols, it is essential to consider the intended purpose. Standardized tests such as the Illinois Agility Test, T-Test, and shuttle run are practical and reliable for comparing athletes across or within groups. However, the scientific literature still presents limited data regarding the use of these tests specifically in women’s futsal, which restricts broader comparisons across studies. This gap is likely due to the general lack of research focused exclusively on women’s futsal players (Barreira et al., 2024).

Intervention studies indicate that agility can be improved in the short term. Patti et al. (2022) evaluated the FIFA 11+ warm-up protocol, a well-known injury prevention program that includes strength, plyometrics, balance, and COD exercises. After 5 weeks of applying FIFA 11+ before training, the experimental group significantly reduced their T-Test time. Although no statistical differences were observed between groups at the end, the findings suggest that both FIFA 11+ and other structured warm-up protocols can enhance agility when performed regularly.

In general, agility performance varies depending on the type of test and the players’ competitive level. Higher-level athletes tend to show shorter completion times, suggesting better agility. This difference likely reflects factors such as more efficient perceptual-motor processes, greater game experience, and more targeted training programs. These findings underscore the importance of systematically including agility training in physical preparation, especially at the youth and developmental levels.

### 3.8 Flexibility

Flexibility is recognized as an important component of physical fitness in women’s futsal, as it directly influences the execution of fast and efficient movements during play. Several recent studies have evaluated flexibility in women’s futsal players across different competitive levels aiming to describe their physical conditioning, compare distinct populations, or analyze the effects of various training interventions. Key studies in this area include those by Alvares et al. (2017), Başkaya et al. (2018), Ceylan et al. (2014), Fiorante and Pellegrinotti (2018), Rocha et al. (2015), and Silva et al. (2017).

All studies included in this review assessed flexibility mainly through the sit-and-reach test using a Wells bench or similar equipment. This consistent methodology ensures basic comparability among results. Alvares et al. (2017) reported an average sit-and-reach score of 30.4 cm in adult players who were runners-up in a regional futsal competition. In college players the studies showed performances of 24.7 Baskaya et al., 2018 and 29.6 cm (Ceylan et al., 2014). Elite professional athletes showed higher performances with a mean flexibility of approximately 36.8 cm at the start of the preparatory period (Rocha et al., 2015). These findings suggest flexibility increases with training experience, peaking in professionally trained athletes, although individual and methodological factors also play a role.

Additionally, Silva et al. (2017) observed interesting interactions between flexibility and other performance variables. They reported a significant positive correlation between initial flexibility and horizontal jump performance after intervention, as well as a negative correlation between initial flexibility and post-intervention body fat percentage. Practically, this indicates athletes with better flexibility tended to benefit more from training—improving jump power and body composition. A plausible explanation is that greater range of motion enables more efficient exercise execution by optimally recruiting muscles and reducing mechanical restrictions, leading to more pronounced physical gains. Although correlations do not imply causality, these results support the notion that flexibility is an integral component of overall athletic performance.

In summary, the evidence reviewed underscores that flexibility allows athletes to achieve larger movement amplitudes in technical gestures, contributing to efficiency in high-speed actions and direction changes (Rocha et al., 2015). Well-designed training programs, even of short duration, can produce significant gains. Given its trainable nature and multifaceted impact on performance, flexibility should be strategically integrated into training programs at all levels of women’s futsal.

## 4 Limitations

Several limitations should be considered when interpreting the findings of this systematic review. First, there was a noticeable disparity in the number of studies addressing each of the physical variables analyzed, resulting in varying degrees of evidence robustness. For instance, external match load and maximal oxygen consumption (VO_2_max) were widely investigated, whereas neuromuscular capacities such as strength and agility received considerably less attention in the literature on women’s futsal players. Second, the instruments, tests, and data collection protocols varied across studies, limiting direct comparability of results. In some cases, this methodological heterogeneity hindered more precise and generalizable interpretations. An additional limitation is the overreliance on simulated and non-competitive match data. These settings often lack the psychological and tactical stressors present in official matches, potentially underestimating true internal and external load responses. Future studies should prioritize data collection in official match environments to enhance the real-world applicability of results. Furthermore, although several studies included biochemical markers such as cortisol and CK, there was limited effort to contextualize these values in terms of athlete monitoring or recovery planning. This gap presents an opportunity for research to bridge physiology and practice more effectively. Other relevant limitations include the absence of studies directly comparing players across different competitive levels and the scarcity of longitudinal investigations tracking physical adaptations throughout the season. These gaps highlight critical areas for future research, underscoring the need for more comprehensive, methodologically consistent studies that reflect the realities of official competition and provide deeper insights into the physical demands and development of women’s futsal athletes.

## 5 Conclusion

This systematic review highlights the high physiological demands and diverse physical profiles of women’s futsal players, reinforcing the need for tailored and evidence-based training strategies. Across studies, players displayed significant cardiovascular and neuromuscular loads, particularly in the first half of matches, with fatigue-related declines observed in the second half.

Recent studies have shown that high-intensity matches lead to increased levels of lactate and creatine kinase (CK), reflecting considerable metabolic and muscular stress that can last for 48–96 h. This evidence highlights the importance of allowing extended recovery periods for players.

From a practical standpoint, coaches and sport scientists should prioritize high-intensity intermittent training, structured aerobic conditioning, and recovery planning informed by physiological monitoring. Sprint and agility training also appear to be particularly relevant, as they are responsive to intervention and correlate with competitive level.

Moving forward, research must expand into longitudinal and ecologically valid designs, focusing on official competitions, inter-positional comparisons, and the integration of physiological, biochemical, and contextual variables. A deeper understanding of real-match demands and individualized responses will enable the development of more precise training models, ultimately enhancing performance and player health across all competitive levels.
